# Robertmurraya beringensis Causing Late-Onset Sepsis in Neonates: A Case Series of a Hitherto Unreported Organism

**DOI:** 10.7759/cureus.88537

**Published:** 2025-07-22

**Authors:** Aayushi Singh, Rema Nagpal, Sujata Deshpande, Deepa Devhare, Vrushali H Thakar, Aishwarya Babu, Pradeep K Suryawanshi

**Affiliations:** 1 Neonatology, Bharati Vidyapeeth (Deemed to be University) Medical College, Pune, IND; 2 Microbiology, Bharati Vidyapeeth (Deemed to be University) Medical College, Pune, IND

**Keywords:** case-series, gram-positive organisms, late-onset sepsis, maldi-tof-ms test, preterm neonates, robertmurraya beringensis

## Abstract

Late-onset sepsis is an important cause of mortality and morbidity in neonates. Gram-positive organisms are increasingly being recognized as causing clinically significant sepsis.* Bacillus beringensis* was first reported as a novel species of the genus *Bacillus* and has not been reported to cause clinically significant infections in humans. This organism was reclassified as *Robertmurraya beringensis *in 2020, following taxonomic changes. We report six neonates, all of whom developed sepsis with this organism. In our patients, the organism was identified with matrix-assisted laser desorption ionization time-of-flight mass spectroscopy (MALDI-TOF-MS) test and revealed a mild-to-moderate virulence pattern. The neonates had predominantly gastrointestinal symptoms, with blood culture positivity in five neonates, and one neonate had the organism in the cerebrospinal fluid. The clinical outcomes were favorable in all the neonates, but one neonate died due to unrelated complications. With the significant emergence of multidrug-resistant organisms and the increasing survival of smaller neonates, this organism may become more significant as a causative agent in NICUs.

## Introduction

Neonatal sepsis is an important cause of morbidity and mortality among neonates, particularly in low- and middle-income countries (LMICs). A systematic review on the global incidence and mortality of neonatal sepsis reported a 1.8-fold higher incidence in middle-income countries, and 3.5-fold higher incidence in low-income countries, with an overall mortality of 17.6% [1]. While both Gram-positive and Gram-negative organisms are known to cause neonatal sepsis, Gram-negative infections predominate in LMICs [2,3]. The Delhi Neonatal Infection Study (DeNIS collaboration, India) attributed 24% of neonatal deaths to sepsis [2]. Late-onset sepsis (LOS) is, conventionally, defined as the onset of sepsis occurring at or beyond 72 hours of life [4]. Among neonates, the National Institute of Child Health and Human Development (NICHD) Neonatal Research Network (NRN) (2002) reported that 70% of first episode LOS was caused by Gram-positive organisms [5], while the DeNIS collaborators [2] found that only 36% LOS infections were caused by Gram-positive organisms among neonates in India.

Among humans, organisms from the *Bacillus* species are opportunistic pathogens in immunocompromised patients, including neonates. This genus, one of the largest and most diverse bacterial genera, with approximately 241 species, is rod-shaped, endospore-forming, aerobic and facultative anaerobic, Gram-positive bacteria, and ubiquitously found in the environment [6,7]. The clinically significant infections caused by organisms from the genus *Bacillus* include *Bacillus anthracis* (causing anthrax) and *Bacillus cereus* (causing food poisoning), while other subspecies such as *Bacillus subtilis* and *Bacillus licheniformis* have been occasionally reported [6,8-10]. *Bacillus beringensis* was first presented as a novel species of the genus *Bacillus* in 2010 [11]. In 2020, following large taxonomic changes, the genus *Bacillus* was reclassified, and multiple novel genera were created, of which the genus “*Robertmurraya*” was one such, named after the Canadian microbiologist Dr. Robert George Everitt Murray, from the University of Western Ontario, Canada, for his contributions and leadership in the field of bacterial taxonomy [12]. We could not find any clinical case reports of patients, whether adult, pediatric, or neonatal, with either *Bacillus beringensis* or the subsequently reclassified *Robertmurraya beringensis*. This case series has been recently presented as a “poster” at the 18th South NEOCON and the 25th KARNEOCON (Karnataka State Neonatal Conference, India) held on June 6-8, 2025.

In this report, we describe six neonates, five of whom had blood cultures that isolated the organism *Robertmurraya beringensis*, while in one neonate, the organism was isolated from the cerebrospinal fluid (CSF). We outline the clinical presentation, laboratory features, treatment, and outcomes in these preterm neonates with the previously unreported *Robertmurraya beringensis*.

## Materials and methods

Case series

This report has received ethical approval from the Institutional Ethics Committee (BVDUMC/IEC/82/24-25), and the need for informed consent was waived due to the retrospective nature of the study. All data were anonymized, and no personal identifiers were collected.

The neonates included in this retrospective case series, collected from a single center, were those who had a conventional blood culture that grew Gram-positive bacilli, later confirmed to be *Robertmurraya beringensis* on matrix-assisted laser desorption ionization time-of-flight mass spectroscopy (MALDI-TOF-MS). All other culture-positive neonates were excluded. Overall, the cases have been described as neonates with “definite” and “probable” *Robertmurraya beringensis* sepsis. Cases 1-6 are labelled as “definite *Robertmurraya beringensis*” sepsis based on their culture positivity and positivity on MALDI-TOF-MS test; while cases 7-11 are labelled as “probable *Robertmurraya beringensis* sepsis,” in view of their very similar organisms on automated blood culture, similar colony morphology, Gram stain features, and clinical presentation. However, these cases (cases 7-11) did not have a MALDI-TOF-MS test sent. The details have been mentioned below. We have utilized the “Preferred Reporting Of CasESeries in Surgery (PROCESS) guidelines” for reporting case series for this publication [13]. 

**Figure 1:**
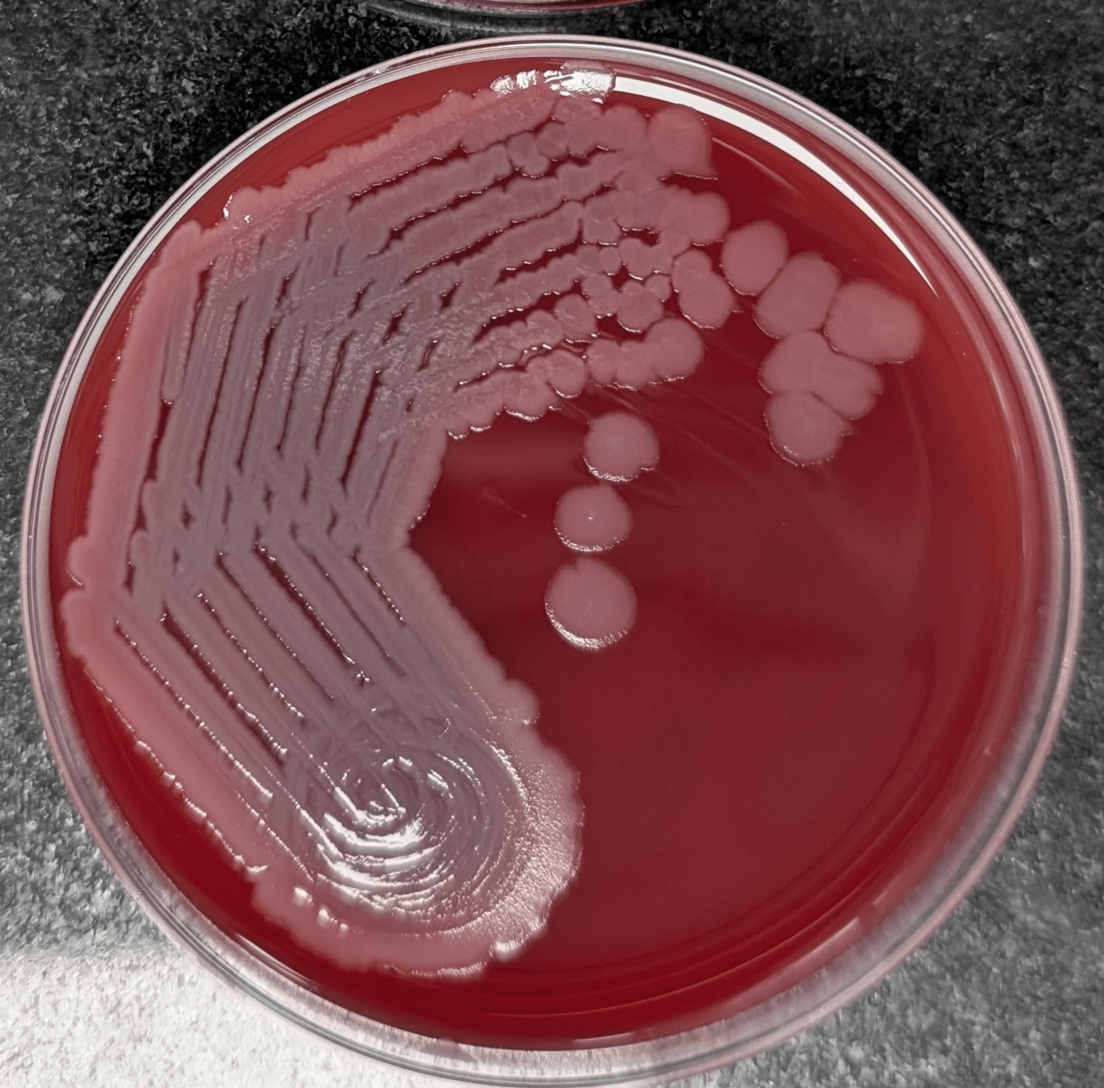
Blood agar shows gray-white nonhemolytic colonies.

We describe six neonates (cases 1-6) who were diagnosed as *Robertmurraya beringensis* sepsis in a level III NICU of a tertiary care hospital in the Western district of Maharashtra, India. These cases occurred over two time periods (cases 1-2 presented in June 2024; cases 3-6 in January 2025), while no cases were reported in the interim. These neonates grew “Gram-positive aerobic spore-bearing bacilli” in the automated blood culture system BACTEC FX40 (Becton Dickinson, East Rutherford, NJ). Once the blood culture flagged positive, it was sub-cultured on 5% sheep blood agar (HiMedia, Mumbai, India) and MacConkey Agar (HiMedia, Mumbai, India). After overnight incubation, the colonies on blood agar appeared as large, grey, white, non-hemolytic, moist colonies with irregular margins, with no growth on MacConkey Agar (Figure 1).

**Figure 2:**
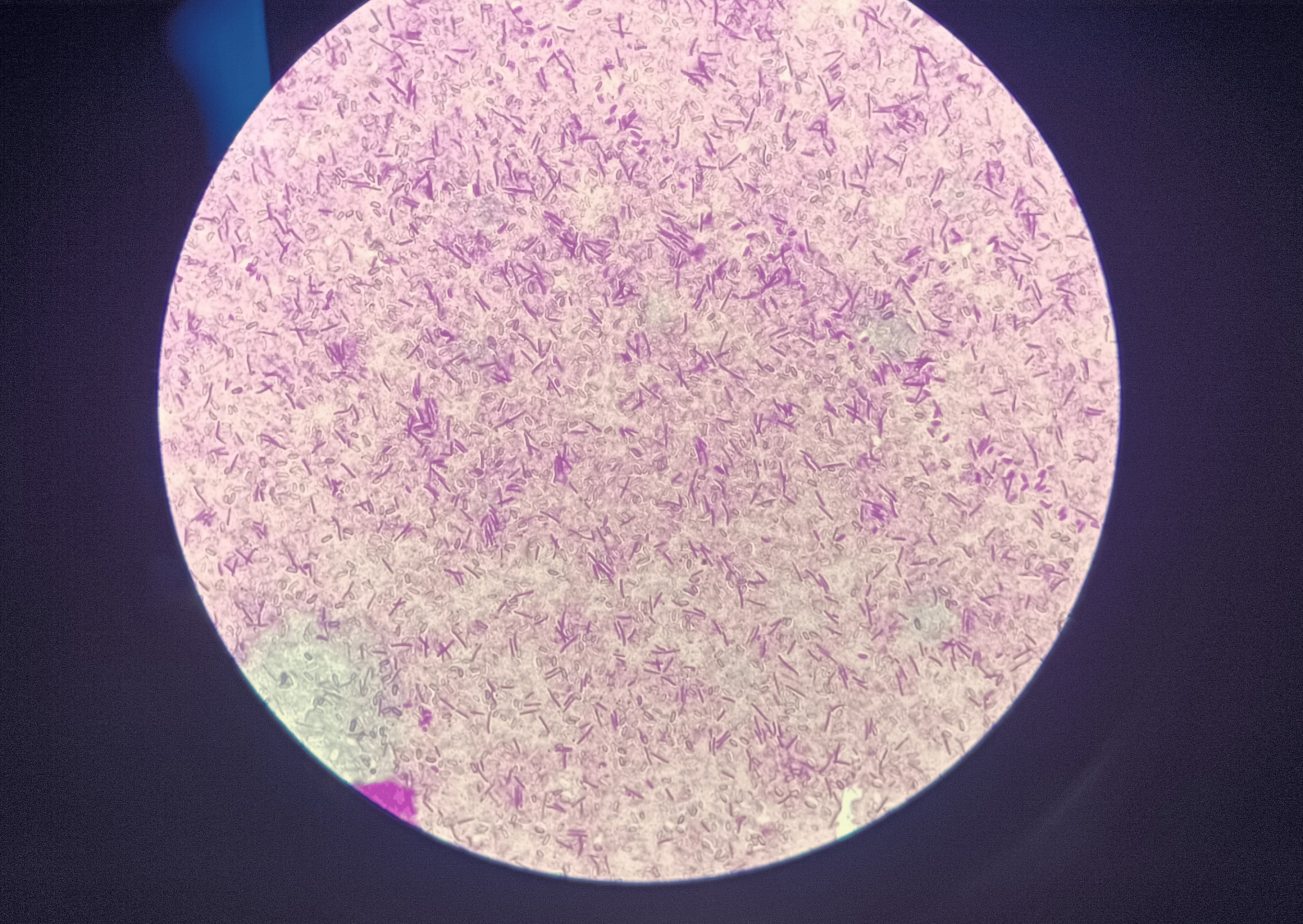
Gram stain shows Gram-positive spore-bearing bacilli.

A Gram stain of these colonies showed large Gram-positive spore-bearing bacilli (Figure 2).

**Figure 3:**
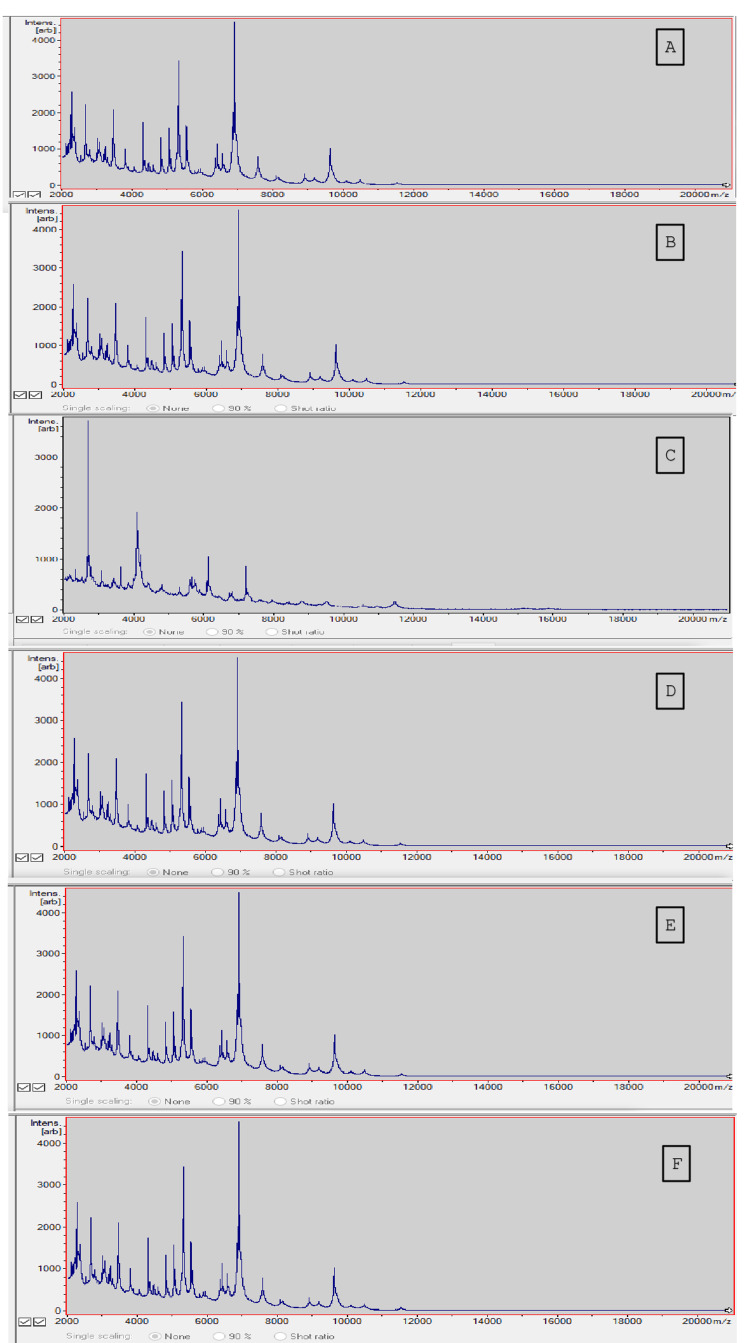
Graphic images of matrix-assisted laser desorption ionization time-of-flight mass spectroscopy (MALDI-TOF-MS) of Robertmurraya beringensis (A-F represent cases 1 to 6, respectively), showing the spatial localized information of the analytes after mass spectroscopic measurement. Images taken from the MALDI Biotyper Sirius-Bruker (Bruker Daltonics GmbH, Bremen, Germany). X-axis represents mass-to-charge ratio (m/z); y-axis represents abundance of ion intensities (depicted as “intensity in arbitrary units” or “intens.[arb]”). Image courtesy: Dr. Sampada Patwardhan, MD

The preliminary biochemical reactions showed them to be catalase and oxidase-positive. Further identification was done using the Bruker MALDI-TOF-MS system (MALDI Biotyper Sirius-Bruker, Bruker Daltonics GmbH, Bremen, Germany). A pure growth of the organism was sent for MALDI-TOF-MS. The sample preparation, sample fixation, ionization, acceleration, detection, and spectrum analysis were done as per the manufacturer's instructions. The methodology behind the test includes fixation in a crystalline matrix on a target plate, and bombardment by laser, followed by the sample molecule vaporization and ionization. The charged particles move after applying a high voltage, and their time of flight is analyzed. The score identification is calculated using the database. In our neonates, the scores were >2 in one neonate, between 1.7 and 1.97 in three neonates, and >1.5 in two neonates. The graphical representation of the MALDI-TOF-MS image is depicted in Figure 3, and details have been explained later in the “Discussion” section.

There are no specific Clinical and Laboratory Standards Institute (CLSI) guidelines for antimicrobial susceptibility testing of this organism, but the microbiology team performed antibiotic-sensitivity testing using the Muller-Hinton Agar by Kirby-Bauer disc diffusion method for Penicillin, Ceftriaxone, Erythromycin, Clindamycin, Tetracycline, Meropenem, Vancomycin, Ciprofloxacin, Piperacillin-Tazobactam, and Gentamycin, to guide the clinical team for treatment. The organism was found to be pan-susceptible to these antibiotics.

Additionally, we provide a brief overview of the data of five more neonates (cases 7-11), described as “probable” *Robertmurraya beringensis* sepsis, who appeared in a cluster along with the index case (case 1) in the first outbreak (June 2024), all within a span of eight days from the index case. In view of similar colony morphology, Gram stain appearance, and preliminary biochemical reactions, identical growth on BACTEC, and a comparable clinical presentation with abdominal symptoms, the clinical and microbiology teams had a strong suspicion of the organism being the same. However, the MALDI-TOF-MS was not performed in the remaining cases (cases 7-11) presenting in June 2024. The clinical presentation of all these cases has been tabulated in Table 1 (as “definite” and “probable” *Robertmurraya beringensis* sepsis), while the laboratory parameters have been presented in Table 2. The reference range for absolute neutrophil counts [14,15] and CSF cell count [16] have been taken from previously published literature.

**Table 1:** Clinical characteristics of neonates with Robertmurraya beringensis sepsis CPAP: continuous positive airway pressure; CS: cesarean section; DOL: day of life; EBM: expressed breast milk; GA: gestational age; IVF: in vitro fertilization; IVH: intraventricular hemorrhage; LONS: late-onset neonatal sepsis; PDHM: pasteurized donor human milk; PICC: peripherally inserted central catheter; PIH: pregnancy-induced hypertension; PV leak: per vaginal leak; RDS: respiratory distress syndrome; TPN: total parenteral nutrition; UAC: umbilical arterial catheter; UVC: umbilical venous catheter

	Case 1	Case 2	Case 3	Case 4	Case 5	Case 6	Case 7	Case 8	Case 9	Case 10	Case 11
Type of sepsis	“Definite” *Robertmurraya* Sepsis						“Probable” *Robertmurraya* sepsis				
Month of presentation	Jun 2024	Jun 2024	Jan 2025	Jan 2025	Jan 2025	Jan 2025	Jun 2024	Jun 2024	Jun 2024	Jun 2024	Jun 2024
Place of birth (Outborn/inborn)	Outborn	Outborn	Outborn	Outborn	Inborn	Outborn	Outborn	Outborn	Outborn	Inborn	Inborn
Mode of delivery	CS	CS	CS	CS	CS	CS	CS	CS	CS	CS	CS
GA at birth (weeks)	33+4	28+0	28+1	36+3	30+4	32+1	27+0	27+0	27+0	39+0	31+2
Birth weight (g)	1870	1185	1140	2100	930	1630	990	860	860	2750	1110
Gender	Male	Male	Male	Male	Male	Female	Male	Female	Male	Male	Male
Maternal risk factors	PV leak < 12 hours	IVF conception	PIH, hypothyroidism	IVF conception, twin gestation	PIH, hypothyroidism	Severe PIH Oligo-hydramnios	Multiple gestation	Multiple gestation	Multiple gestation	Nil	PIH
Associated comorbidity	Prematurity	Prematurity RDS	Prematurity, RDS	Congenital diaphragmatic hernia (CDH)	Prematurity, cholestasis, *Klebsiella* and *Serratia* *marcescens* sepsis	Prematurity RDS	Prematurity RDS	Prematurity RDS	Prematurity RDS, pulmonary hemorrhage	Nil	Prematurity
Day of life at admission to NICU	1	1	1	38	1	2	1	1	1	1	1
Day of life of symptom onset	5	4	2	42	40	2	4	4	4	2	4
DOL of RM blood culture positivity	7	4	4	42	53	4	4	4	4	4	4
Symptoms/signs at sepsis onset	Vomiting apnea	Abdominal distension, bilious gastric aspirate	Hyperglycemia, abdominal distension, bilious gastric aspirates, metabolic acidosis	Fever	Abdominal distension, ascites, deranged liver enzymes, meningitis	Abdominal distension, non-bilious vomiting	Persistent unexplained metabolic acidosis	Persistent unexplained metabolic acidosis	Persistent unexplained metabolic acidosis	Fever, abdominal distension, meningitis	Altered gastric aspirates, fever, abdominal distension
Ventilation status at time of infection	CPAP	Invasive	Invasive ventilation for RDS	Invasive ventilation for CDH	Invasive ventilation for sepsis	Non-invasive ventilation	Invasive ventilation for RDS	Invasive ventilation for RDS	Invasive ventilation for RDS	Nil	Non-invasive ventilation
Central lines UVC/UAC/PICC at sepsis onset	Nil	UAC, UVC	UAC, UVC	Nil (placed after surgery)	PICC	PICC	UVC, UAC	UV, UAC	UVC, UAC	Nil	PICC
TPN (yes/no)	Yes	Yes	Yes	Yes	Yes	Yes	Yes	Yes	Yes	Yes	Yes
Feed volumes at sepsis onset	38 mL/kg/day	20 mL/kg/day	10 mL/kg/day	NBM	NBM	20 mL/kg/day	28 mL/kg/day	28 mL/kg/day	12 mL/kg/day	NBM	60 mL/kg/day
Type of milk	EBM	PDHM	EBM =/PDHM	NIL	EBM	EBM/PDHM	EBM/PDHM	EBM/PDHM	EBM/PDHM	NIL	PDHM
MALDI-TOF score	1.58	1.54	2.13	1.97	1.79 (in CSF)	1.7	Not done	Not done	Not done	Not done	Not done
Management	Gentamicin Meropenem × 10 days	Gentamicin Meropenem × 7 days; Piperacillin, Tazobactam × 10 days; Inotropes for 12 hours	Ampicillin Gentamicin × 1 day; Meropenem × 10 days	Meropenem Vancomycin × 7 days	Meropenem-Levofloxacin-Vancomycin; blood, platelet transfusions	Gentamicin meropenem × 14 days	Gentamicin × 2 days; Meropenem × 10 days; Piperacillin-Tazobactam × 4 days	Gentamicin × 2 days; Meropenem × 10 days; Piperacillin-Tazobactam × 4 days	Gentamicin × 2 days; Meropenem × 5 days; Piperacillin-Tazobactam × 10 days; blood transfusion; inotropes	Gentamicin × 3 days; Meropenem × 21; days; Colistin × 21 days	Gentamicin Meropenem × 10 days
Morbidity at discharge (CLD, ROP, IVH, PVL)	Nil	Nil	CLD (Grade II)	Nil	Cystic PVL (antecedent to sepsis)	Nil	Bilateral Grade 1 IVH	Bilateral Grade 1 IVH	Bilateral Grade 1 IVH, CLD	Nil	Nil
Outcomes	Discharged on DOL 21	Discharged on DOL 57	Discharged on DOL 88	Discharged on DOL 60	Death on DOL 106 of unrelated complications	Discharged on DOL 22	Discharged on DOL 90	Discharged on DOL 90	Discharged on DOL 90	Discharged on DOL 24	Discharged on DOL 35

**Table 2:** Laboratory and cerebrospinal fluid characteristics of neonates with Robertmurraya beringensis sepsis

Case	Nadir WBC count (per mm^3^) after disease onset; normal WBC counts = 4500-11,000 per mm^3^	Absolute neutrophil count (mm^3^) after disease onset [14,15]	Nadir platelet count (lakh/μL) after disease onset (normal = 1.5-4.5 lakh/μL)	C-reactive protein (mg/dL) (normal = 0-5 mg/dL)	CSF cell count (cells/μL) (normal = 0-32 cells/μL)	CSF biochemistry (normal sugar = 55-105 mg/dL, normal protein = 65-150 mg/dL)	CSF culture
“Definite” *Robertmurraya* sepsis							
Case 1	8500	3300	180,000	1	6 cells	Sugar - 49, protein - 78	No growth
Case 2	20,400	13260	159,000	2.7	25 cells, predominant neutrophils	Sugar - 51, protein - 112.2	No growth
Case 3	13,100	7598	156,000	3.6	8 cells	Sugar - 49, protein - 107.6	No growth
Case 4	14,400	5040	284,00	28	Not sent		Not sent
Case 5	3400	2482	17,000	137.6	1920 cells, 95% neutrophils	Sugar - 25, protein - 156	Robertmurraya beringensis
Case 6	19,600	11,956	309,000	2.71	3 cells	Sugar - 63, protein - 112	No growth
“Probable” *Robertmurraya* sepsis							
Case 7	4200	1218	122,000	1.3	1 cell	Sugar - 67, protein - 98	No growth
Case 8	3200	928	113,000	2.8	1 cell	Sugar - 56, protein - 112	No growth
Case 9	3800	532	130,000	1.6	1 cell	Sugar - 69, protein - 133	No growth
Case10	4100	2050	41,000	177.9	150 cells, 65% neutrophils	Sugar - 41, protein - 152	No growth
Case11	6600	3432	75,000	6.4	6 cells	Sugar - 71, protein - 126	No growth

Five of the reported six neonates with proven *Robertmurraya sepsis* were out-born, all were preterm neonates, born by caesarean section, with gestational ages ranging from 28+0 to 36+3 weeks. Five of the six neonates became symptomatic within the first week of admission with a clinical suspicion of sepsis. In five of the neonates (cases 1, 2, 3, 5, and 6), the predominant symptoms were gastrointestinal (vomiting, abdominal distension, and bilious aspirates), while one neonate (case 5) also had meningitis. The blood cultures of all these patients grew aerobic Gram-positive spore-bearing bacilli in the automated blood culture system BACTEC FX 40. All the neonates had a favorable clinical course in the NICU; five were discharged, while one neonate (case 5) died 66 days later due to unrelated complications.

Surveillance for the infection source

Since the cases presented in two clusters, in June 2024 and January 2025, with a previously unreported organism, in whom there were no known clinical characteristics, a comprehensive surveillance was carried out, on both occasions, to determine the likely source of the infection. These included (i) neonatal surveillance (blood and CSF cultures for all neonates, urine cultures were sent if the neonate was >7 days of age), stool culture was sent in one neonate (ii) surveillance of health care workers (random hand swabs of doctors, nurses, multipurpose worker staff responsible for environmental cleanliness), (iii) environmental survey - ventilator circuit swabs, ventilator humidifier fluid cultures, cultures from total parenteral nutrition (TPN) fluid and equipment used for preparation of TPN (surface swabs from laminar flow cabinet before and after cleaning), culture of water sources including tap water, cultures of intravenous fluids (glucose, saline), random cultures of washbasins in NICU, cultures of breast milk, milk-containers, echocardiography jelly, cultures from the neonate’s bed, and (iv) observation of the cleaning/disinfection procedures including a critical evaluation of adherence to existing protocols for cleaning and disinfection of equipment by the infection control nurse/department. This extensive surveillance did not reveal any source of infection in either of the episodes. Appropriate isolation precautions were taken to prevent the spread of infection amongst other admitted neonates in both instances. No swabs were taken from the non-septic neonates admitted to the NICU. However, the other neonates admitted to the NICU, during both periods, were kept under observation for any symptoms that could suggest a similar infection.

## Results

Cases with “definite” *Robertmurraya beringensis* sepsis

Six neonates (two neonates in June 2024, four neonates in January 2025) were diagnosed with *Robertmurraya beringensis* sepsis following MALDI-TOF-MS, and their presentation, management, and outcomes are described below:

Case 1

A 33+4 gestation out-born preterm neonate, with birth weight (BW) 1870 g, was referred to the NICU (in June 2024) on day of life (DOL) 1 for respiratory distress and prematurity. The baby was managed initially with continuous positive airway pressure (CPAP), TPN, and enteral feeds were commenced on day 1. The neonate developed vomiting, apnea, and required escalation of respiratory support on DOL 5. The automated blood cultures grew “Gram-positive aerobic spore-bearing bacilli,” and for microorganism identification, a MALDI-TOF-MS was sent, which revealed an isolate of *Robertmurraya beringensis* (score 1.58). The neonate was initially administered Gentamicin (as per unit protocol), but on subsequent clinical deterioration and pending organism identification, Meropenem was commenced. Repeat blood cultures (on DOL 7 and 14) remained positive, since the neonate remained clinically stable, no further change of antibiotics was considered. A repeat blood culture on day 18 was negative. The infant was discharged on DOL 21 with no significant morbidities. 

Case 2

A 28+0 week out-born preterm neonate, BW 1185 g, was admitted (in June 2024) on DOL 1 for prematurity and respiratory distress syndrome (RDS). The baby initially required invasive ventilation, TPN, and umbilical catheters. The baby worsened on DOL 4 with abdominal distension and bilious aspirates, and on clinical suspicion of sepsis, commenced on Gentamicin and Meropenem. The baby required inotropic support for 12 hours. The blood cultures (sent on DOL 4) grew Gram-positive bacilli; MALDI-TOF-MS confirmed *Robertmurraya beringensis* (score 1.54), while CSF was sterile. Repeat blood cultures on DOL 10 showed a recurrent growth, and antibiotics were changed to Inj. Piperacillin-Tazobactam. The baby subsequently improved and was discharged on DOL 57 with no significant morbidities.

Case 3

A 28+1 week out-born preterm neonate, BW 1140 g, was admitted on DOL 1 with prematurity and RDS, required invasive ventilation, surfactant administration, TPN, and central lines. Antibiotics were initially commenced empirically due to unexplained prematurity with meconium-stained liquor; however, the admission blood culture was sterile. On DOL 3, the baby developed hyperglycemia, metabolic acidosis, and associated gastrointestinal symptoms (abdominal distension, bilious aspirate). The blood culture (sent on DOL 4) grew Gram-positive bacilli, while the CSF was sterile. MALDI-TOF-MS confirmed *Robertmurraya beringensis* (score 2.13). The neonate was given Inj. Meropenem and remained stable thereafter. The baby developed grade II bronchopulmonary dysplasia (BPD) [17] and was eventually discharged on DOL-88.

Case 4

**Figure 4:**
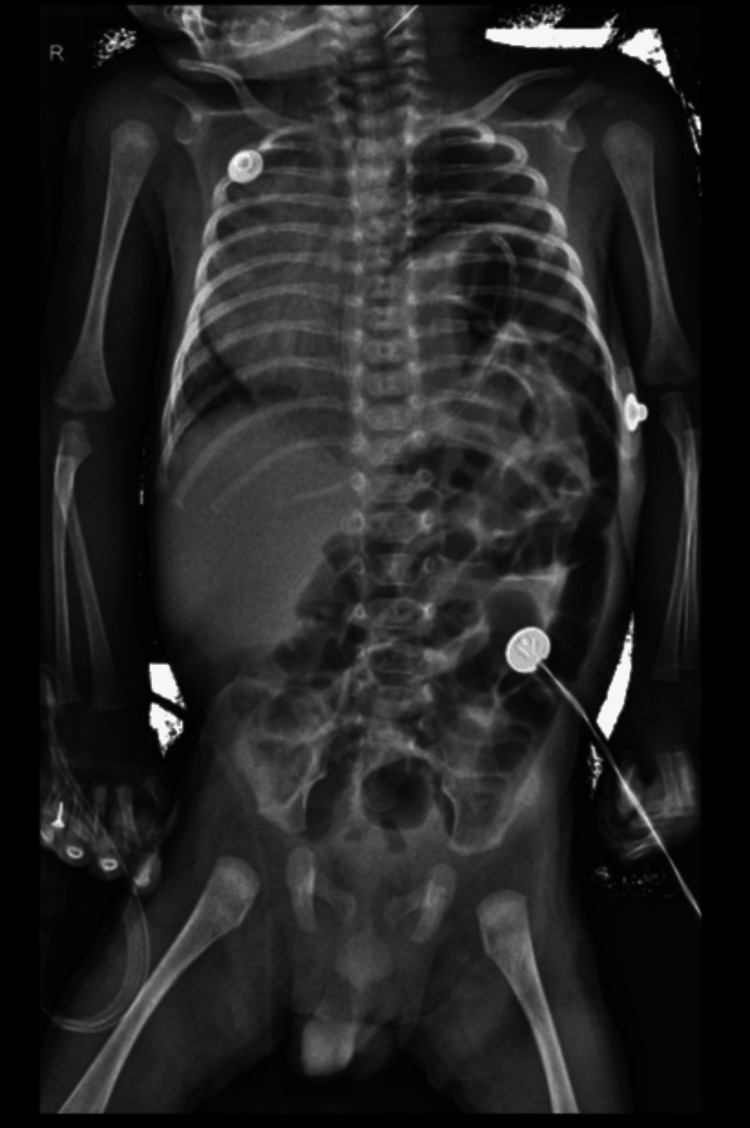
X-ray chest of neonate (case 4) with previously undetected congenital diaphragmatic hernia.

A 36+3 week out-born, late preterm, BW 2100 g, was admitted on DOL 38 with respiratory distress and decreased oral intake. An X-ray revealed a previously undetected left-sided congenital diaphragmatic hernia (CDH) (Figure 4) and required invasive ventilation in view of a high oxygen requirement. CDH repair surgery was done three days after hospitalization. On post-operative day 2, the baby developed two fever spikes (temperature range: 37.8-38.7°C) and was commenced on Inj. Meropenem and Vancomycin. The MALDI-TOF-MS was positive for the organism (score 1.97). Surveillance swabs from the operation theater (OT) were found to be satisfactory, and hence the possibility of contracting infection from there was ruled out. Antibiotics were given for seven days, and the baby improved thereafter and was discharged on DOL 60 with no significant morbidities.

Case 5

A 30+4 week inborn preterm neonate, BW 930 g, had an uneventful initial NICU course, needing non-invasive (NIV) respiratory support till DOL 27. The baby had one episode of “suspect” sepsis on DOL 19, but cultures were negative. On DOL 40, the baby became symptomatic, with abdominal distension and respiratory distress requiring NIV ventilation, and Inj. Meropenem was commenced. The blood culture grew *Klebsiella pneumoniae*, while the infant developed ascites, direct hyperbilirubinemia, significant thrombocytopenia, and required multiple blood and platelet transfusions. Since the baby was clinically deteriorating, a repeat blood culture (seven days later) was sent, which grew *Serratia marcescens*, and antibiotics were appropriately changed as per sensitivity. A further seven days later, the blood culture was positive for Gram-positive aerobic spore bearing bacilli, and CSF (not previously done since the neonate was clinically unstable) revealed evidence of meningitis (total cell count - 1920 cells/μL, with 95% neutrophils, normal range = 0-32cells/μL, CSF protein - 156 mg/dL, CSF sugar - 25mg/dL). Since the automated blood culture system could not identify the organism, MALDI-TOF-MS on the CSF sample was performed, which revealed an isolate of *Robertmurraya beringensis* (score 1.79). The infant variously received multiple antibiotics (Meropenem, Vancomycin, and Levofloxacin), and the meningitis subsequently resolved (repeat CSF showed 10 cells/μL with no growth). Subsequently, the baby had a stormy NICU course, developed significant ascites, direct hyperbilirubinemia, and deranged liver enzymes. The infant was noted to have a cystic periventricular leukomalacia (PVL) antecedent to the sepsis. The baby remained in the hospital for unrelated complications and, unfortunately, died on DOL 106. This death was not attributed to *Robertmurraya beringensis* sepsis since repeat blood and CSF cultures were sterile.

Case 6

A 32+1 week out-born female neonate, BW 1630 g, was admitted on DOL 2 with prematurity, RDS, abdominal distension, and vomiting. The neonate required NIV support, and Inj. Gentamicin was given as per NICU protocol. The initial septic workup and blood cultures were negative. On starting enteral feeds, the neonate had abdominal distension with tenderness. Inj. Meropenem was started, and a lower gastrointestinal dye study (suggested by the pediatric surgery team) was normal. A repeat blood culture on DOL 4 grew Gram-positive bacilli, confirmed by MALDI-TOF-MS as *Robertmurraya beringensis* (score 1.7), while CSF was sterile. The abdominal distension subsequently improved, and feeds were reintroduced, which were tolerated well. The baby was discharged on DOL 22. 

Cases with “probable” *Robertmurraya beringensis* sepsis

The clinical presentation of the five cases, which were diagnosed simultaneously with the index case, has been tabulated in Table 1 under the section “probable sepsis.” All these neonates had “Gram-positive aerobic spore-bearing bacilli” in their VITEK reports. All these neonates presented with abdominal symptoms, and additionally, thrombocytopenia, while one neonate presented with meningitis. These neonates were not diagnosed using MALDI-TOF, but in view of similar colony morphology, Gram stain appearance, identical growth on BACTEC, and a comparable clinical presentation, a strong suspicion of *Robertmurraya beringensis* was considered by the clinical and microbiology teams. We, therefore, present this as additional data for consideration.

Cases 7-9

Triamniotic-trichorionic out-born preterm extremely low birth weight (ELBW) triplet neonates, born at 27+0 weeks, were admitted on DOL 1, for prematurity and RDS. All the neonates required respiratory support, surfactant administration, TPN, and central lines. On DOL 4, all three neonates developed unexplained metabolic acidosis, leukopenia, and neutropenia. The cultures from all three neonates grew “Gram-positive bacilli.” Subsequently, the third triplet developed massive pulmonary hemorrhage and required blood transfusions and inotropic support. The triplets had an uneventful course thereafter and were discharged on DOL 90.

Case 10

A 39-week inborn term neonate was admitted to the NICU on DOL 2 for abdominal distension and delayed passage of meconium. After NICU admission, the neonate had multiple episodes of fever, thrombocytopenia, raised C-reactive protein (177.9 mg/L, reference range 0-5 mg/dL), and raised CSF cell cytology (150 cells/μL, 65% neutrophils, reference range 0-32cells/μL). The neonate was commenced on Inj. Meropenem and Gentamicin, kept nil oral and commenced on TPN. The blood culture showed growth of “Gram-positive bacilli,” while CSF remained sterile. In view of continued persistent fever spikes, Inj. Colistin was added. The neonate responded well thereafter and was given a total duration of antibiotics of 21 days and discharged on DOL 24.

Case 11

A 31+2 week inborn preterm, BW 1110 g, was clinically stable on high flow nasal canula 3L/min, with FiO2 0.21 and on routine supportive preterm care. On DOL 4, the neonate developed gastrointestinal symptoms (abdominal distention, altered brownish gastric aspirates), fever, and thrombocytopenia. Blood culture showed growth of “Gram-positive bacilli,” while CSF was sterile. Antibiotics (Inj. Gentamicin, Meropenem) were given for 10 days. The baby was discharged on DOL 35 with no major morbidities.

## Discussion

We report six neonates who were diagnosed to have *Robertmurraya beringensis* sepsis, which has not previously been reported to cause infection in humans. This organism is a Gram-positive, rod-shaped, endospore-forming, motile bacterium. The genus *Robertmurraya* was created in 2020 from the polyphyletic genus *Bacillus* and was first described by Gupta et al. [12,18]. Genus *Robertmurraya* currently (as of May 2021) includes 10 species, of which *Robertmurraya beringensis*, described in this case series, is one; the other species include *Robertmurraya spiralis*, *Robertmurraya andreesenii*, *Robertmurraya crescens*, *Robertmurraya korlensis*, *Robertmurraya kyonggiensis*, *Robertmurraya massiliosenegalensis*, *Robertmurraya yapensis*, *Robertmurraya dakarensis*, and the uncharacterized *Bacillus* sp. Y1 [12,19]. In 2003, a psychrotolerant (organisms capable of growth close to 0°C but with optimum growth temperature >20°C) *Bacillus*-like strain was isolated from the sea water of the Bering Sea by the Second Chinese National Arctic Research expedition and named *Bacillus beringensis* [11]. In view of the antecedent taxonomy of the genus *Robertmurraya*, we can, perhaps, compare this series of neonates with those infected with *Bacillus* sp. (particularly *Bacillus cereus*), which are, similarly, Gram-positive, endospore-forming, rod-shaped bacteria. As of 2022, 145 cases of *Bacillus cereus* have been reported, with a wide spectrum of clinical presentations in neonates (preterm and term), including sepsis (48% cases), central nervous system infections (25%), respiratory infections (12%), and gastrointestinal infections (4%) [7]. Quite unlike *Bacillus cereus*-infected neonates, who may have devastating intestinal infections and outcomes, our neonates presented with moderate symptoms, predominantly gastrointestinal. Organisms from the genus *Bacillus* are ubiquitously found in dust, air, and water, with sporadic reports of meningitis, bacteremia, respiratory tract infections, and intestinal perforations [7]. The reported growth of *Bacillus beringensis* occurs at 4-42°C (optimum 30-33°C) [11]. The pH and salinity for growth range from 5.5 to 10.5 (optimum pH 6.0-8.0) and 0-8% NaCl (optimum 0-3%)[11]. Members of the genus *Robertmurraya* are aerobic, found in sand soil and human stools, and produce endospores, which have a high resistance to extreme conditions, including heat, cooking, boiling, and high salinity.

Traditionally, the VITEK system is the most commonly used automated identification technique in microbiology laboratories for microbial identification; however, the available VITEK card for Gram-positive bacilli cannot identify *Robertmurraya* species, and hence, the isolate was sent for further testing. MALDI-TOF-MS is a rapid and reliable technique to identify, type, and differentiate closely related biomolecules, including protein sequences, and subsequently compares it to comprehensive large databases of mass spectral fingerprints, to assist in typing of strains of specific genera/species/subspecies/strains [20]. This protein profiling is used reliably to identify closely related species, including *Bacillus* spp. The use of MALDI-TOF-MS has significantly increased over the last few years, since it offers a highly accurate species-level identification in minutes, at relatively low costs, with accuracy that matches or even exceeds conventional identification systems [20]. MALDI-TOF-MS can be used to identify 99.6% isolates at the genus level, and 93.4% isolates to the species level [21]. The MALDI-TOF-MS scoring system compares the sample mass spectrum to the reference mass spectrum in the database, and calculates a score, ranging from zero to three, reflecting the extent of similarity between the sample and the reference spectrum. These scores are used as a tool for interpretation of the reliability for genus and species identification, with scores ≥ 2 considered reliable for species identification, while score values between 1.7 and 2.0 are reliable for identification at the genus level [22]. A mass spectrum image displays mass-to-charge ratio (m/z) on the x-axis, and abundance of ion intensities as “intensity in arbitrary units” or “intens. [arb]” on the y-axis. Each peak on the graph indicates the relevant abundance of the ions at a specific m/z value, with higher peaks suggesting more abundant values. The overall peak pattern creates a unique spectral signature specific to the analyte. 

Identification of *Bacillus* spp. is not as simple as for other bacteria because of the presence of endospores. Endospores have different protein expression compared to vegetative cells, likely leading to inconsistencies in *Bacillus* identification, and fresh cultures of the isolates are preferable for identification [23]. *Bacillus* species, isolated from blood cultures, are often considered a collection contaminant; however, in our case series, since all the neonates were symptomatic and immunocompromised, we processed these isolates as potential pathogens. The early identification of a microorganism facilitates measures to control its spread and helps in understanding the epidemiology of the organism. As can be seen in our series, MALDI-TOF-MS aided us in the timely detection of the pathogenic bacteria, since the automated identification method was unable to identify this organism. However, this could not be undertaken in all the suspected cases in the first outbreak. We, therefore, emphasize the importance of using MALDI-TOF-MS for identifying bacterial infections rapidly, and with greater identification accuracy, with a reduced identification error rate.

The six neonates described in this series with “definite R. beringensis sepsis” were all preterm, predominantly males, all born by caesarean section, exhibited a range of disease manifestations, ranging from mild to moderate virulence patterns. One neonate among the neonates with R. beringensis had the organism in the CSF, but this neonate was also simultaneously being treated for *Klebsiella* and *Serratia* sepsis, and the stormy NICU course could possibly be attributed to those organisms. However, among the neonates in the previous episode of infection (June 2024), one had meningitis, and one extreme preterm had pulmonary hemorrhage. Hematological manifestations in the form of mild-to-moderate thrombocytopenia and absolute neutropenia were seen in a number of the neonates in both clusters. Following the blood culture positivity in our preterm neonates, with a previously unreported organism, and the absence of any antecedent literature concerning antibiotic sensitivity, varied antibiotics, including carbapenems, aminoglycosides, and penicillin analogues, were given, for varying durations, particularly in the earlier episode in June 2024. Literature suggests that *Bacillus cereus* has sensitivity to a number of antibiotics, including aminoglycosides, carbapenems, and vancomycin [7]. Among the cluster of neonates infected in January 2025, the neonates recovered rapidly, barring one neonate with meningitis. In the absence of drug sensitivity patterns, we are unable to make a strong recommendation on the suggested antibiotics for this organism, and this will remain an area of focus in case of a repeat outbreak. We speculate that this organism may likely have a similar presentation across different gestational ages (i.e., presentation with abdominal symptoms), but the disease expression may differ depending on the extent of prematurity, and possibly bacterial load. The likely focal reservoir of *Robertmurraya beringensis* could not be determined in spite of our extensive epidemiological investigation. The common interventions, like TPN and central lines, were repeatedly investigated to rule them out as infection sources. Assessment of the origin of infections due to an organism like *Robertmurraya beringensis*, which is so widely disseminated in the environment, is difficult and may not yield an obvious source. Since members of the genus *Robertmurraya* are found in soil and human stools, we speculate that contamination from external sources, such as soil, etc., was a likely cause. The available data, consequent to our surveillance, does not enable us to suggest the possible mode of transmission or the source of this outbreak. We suggest that the possibility of the organism being a contaminant is unlikely, since the neonates were symptomatic, with similar clinical presentation, identical growth in the VITEK blood culture and MALDI-TOF-MS, with the outbreak noted in two different time periods, with no cases seen in the interim.

There are some limitations to this study. In view of this organism being previously unreported, this has been presented as a case series. However, to determine the true incidence of the disease, it would be useful to send MALDI-TOF-MS in all clinically septic, though VITEK culture-negative neonates, with a similar clinical and microbiological profile (on Gram stain, BACTEC system). Besides, there are no specific CLSI guidelines for antimicrobial susceptibility testing of this organism. We, therefore, remain unable to make a strong recommendation on the suggested antibiotics for this organism, and as mentioned earlier, this will remain an area of focus in case of a similar outbreak. We also suggest that the above-described clinical practice may limit the generalizability of our treatment results to other settings.

To the best of our knowledge, this is the first reported case series of *Robertmurraya beringensis*, causing a clinical infection in humans, and diagnosed by MALDI-TOF-MS molecular assay.

## Conclusions

We report the clinical presentations of a previously unreported organism, *Robertmurraya beringensis*, in the NICU, primarily affecting preterm neonates, and manifesting with gastrointestinal symptoms. The organism exhibited a range of virulence, ranging from mild to moderate, but neonates, mostly, had a stable clinical course, had an adequate response to antibiotics, and one neonate died due to unrelated complications. The automated microbial identification system is not effective in identifying *Robertmurraya* species, and MALDI-TOF-MS testing should be utilized for greater accuracy in diagnosis. The persistence of bacteremia despite the use of broad-spectrum reserve antibiotics indicates the need to differentiate from previously unreported infections, while excluding the possibility of the organism being a contaminant. With improved survival of smaller neonates, such an unreported organism may become more prevalent and require improved diagnostic tests for identification. Future outbreaks of this organism will need to focus on disease expression and antibiotic susceptibility patterns to determine the best antimicrobial coverage for such neonates.
